# Acute Effects of Albuterol on Ventilatory Capacity in Children with Asthma

**DOI:** 10.3390/pediatric16010005

**Published:** 2024-01-05

**Authors:** Michael W. H. Wong, Lung-Chang Chien, Dharini M. Bhammar

**Affiliations:** 1 Department of Kinesiology and Nutrition Sciences, School of Integrated Health Sciences, University of Nevada Las Vegas, Las Vegas, NV 89154, USA; michael.wong@unlv.edu; 2 Department of Epidemiology and Biostatistics, School of Public Health, University of Nevada Las Vegas, Las Vegas, NV 89154, USA; lung-chang.chien@unlv.edu; 3 Center for Tobacco Research, Department of Internal Medicine, College of Medicine, The Ohio State University, Columbus, OH 43210, USA

**Keywords:** maximal exercise, cardiopulmonary exercise test, breathing limitation, bronchodilation, pediatric, dyspnea

## Abstract

Background: Children with asthma may have a reduced ventilatory capacity, which could lead to symptoms and early termination of a cardiopulmonary exercise test (CPET). The purpose of this study was to examine the effects of short-acting beta agonist (albuterol) administration on estimated ventilatory capacity in children with asthma. Methods: Fifteen children (eleven boys, 10.6 ± 0.9 years) completed spirometry at baseline, after 180 µg of albuterol, and after the CPET in this cross-sectional study. Ventilatory capacity was calculated from forced vital capacity (FVC) and isovolume forced expiratory time from 25 to 75% of FVC (isoFET_25–75_) as follows: FVC/2 × [60/(2 × isoFET_25–75_)]. Differences in outcome variables between baseline, after albuterol administration, and after the CPET were detected with repeated measures mixed models with Bonferroni post hoc corrections. Results: Estimated ventilatory capacity was higher after albuterol (68.7 ± 21.2 L/min) and after the CPET (75.8 ± 25.6 L/min) when compared with baseline (60.9 ± 22.0 L/min; P = 0.003). Because forced vital capacity did not change, the increased ventilatory capacity was primarily due to a decrease in isoFET_25–75_ (i.e., an increase in mid-flows or isoFEF_25–75_). Conclusion: Albuterol administration could be considered prior to CPET for children with asthma with relatively well-preserved FEV_1_ values to increase ventilatory capacity pre-exercise and potentially avoid symptom-limited early termination of testing.

## 1. Introduction

Cardiopulmonary exercise testing (CPET) is a gold-standard diagnostic tool to assess functional capacity [1]. The presence of ventilatory limitations or reduced breathing reserve could limit the ability of patients with asthma to provide maximal effort during testing leading to premature test termination or a symptom-limited exercise test and consequent difficulty in accurately assessing functional capacity or cardiorespiratory fitness [2]. Short-acting β_2_-adrenoceptor agonist inhalers such as albuterol (brand names Ventolin, ProAir, and Salbutamol) could increase maximal and mid-expiratory flows via bronchodilation [3,4], which has the potential to increase breathing reserve and reduce ventilatory limitations during exercise in children with asthma. While the American Thoracic Society guidelines recommend that patients remain on their prescribed medications before CPET if the purpose of the test is to assess functional capacity or cardiorespiratory fitness [5], it is not clear if albuterol is routinely administered before CPET for children with asthma. Studying the effects of albuterol on ventilatory capacity could offer important insights into whether albuterol should be administered before CPET if the goal is to determine functional capacity or to diagnose deconditioning/reduced cardiorespiratory fitness.

Traditionally, maximum voluntary ventilation (MVV) has been used as a surrogate of ventilatory capacity [6]. MVV is either measured directly with a breathing maneuver or indirectly as the product of forced expiratory volume in 1 s (FEV_1_) and an appropriate factor such as 37.5 [7], but there are limitations in this approach. For example, during direct MVV testing, participants breathe with high frequency and low tidal volumes (V_T_) and at higher lung volumes nearing total lung capacity, which is a rare pattern of breathing during exercise, which can lead to the overestimation of ventilatory capacity [8,9]. Indirectly obtaining MVV via calculation with FEV_1_ has shown to produce significantly higher values when compared with direct measurement of MVV in children, which can also overestimate ventilatory capacity [10].

An alternate estimate of ventilatory capacity described by Babb and Rodarte [11] and Bartlett et al. [12] is based on measurements from the mid-expiratory portion of the flow–volume curve [13]. Forced expiratory time between 25 and 75% of FVC (isoFET_25–75_; see Section 2.3 for description of isovolume) is doubled to represent the minimum achievable total respiratory cycle time, which can offer an estimate of maximal breathing frequency (*f*_B_, 60 s/(2 × isoFET_25–75_)). Ventilatory capacity is the product of the estimated maximal *f*_B_ and estimated maximal tidal volume (FVC/2) because tidal volume typically approaches 50% of forced vital capacity (FVC) during maximal-intensity exercise [14]. Estimating ventilatory capacity using this method better mimics breathing patterns during exercise when compared with the MVV maneuver [8,9]. Therefore, estimating ventilatory capacity using the mid-expiratory portion of the flow–volume curve may have higher physiological relevance compared with MVV-derived ventilatory capacity.

The effects of albuterol on preventing exercise-induced bronchoconstriction and treating acute bronchospasm among asthmatic individuals are well described in the literature [3,15,16,17]. Recently, Wilhite et al. [13] reported a 15.8% increase in estimated ventilatory capacity after administration of albuterol among nonasthmatic children with and without obesity. Considering that albuterol induces an increase in ventilatory capacity even among children without asthma, it could be useful to understand whether albuterol can produce an increase in ventilatory capacity among children with asthma who may stand to benefit from the improved ventilatory capacity in the context of exercise. However, little is known about the effects of albuterol on estimated ventilatory capacity in children with asthma. Therefore, the purpose of this study was to examine the effects of albuterol on estimated ventilatory capacity. We hypothesized that albuterol administration would increase estimated ventilatory capacity. These data were collected as part of a larger project comparing the effects of albuterol vs. high-intensity interval warm-up exercise on exercise-induced bronchoconstriction during constant work rate cycling exercise in children with asthma. Some data in this paper have been previously published in abstract form [18].

## 2. Materials and Methods

The study was approved by the University of Nevada, Las Vegas IRB (#1131374) and Sunrise Hospital & Medical Center IRB (#17-048). All study procedures and protocols were explained in detail to children and their parents. Children provided assent, and parents provided written informed consent. Participants were recruited from a pediatric pulmonology clinic in Las Vegas, NV, USA from September 2018 to March 2020. We recruited parents with the help of flyers posted at the clinic and by contacting parents of potentially eligible children screened through the medical record for inclusion/exclusion criteria with permission from the treating physician. Inclusion criteria for the study were 8–12 years of age and a physician diagnosis of asthma. On the first study visit, children with help from their parents completed a self-report medical history form [19] and physical activity readiness questionnaire [20]. Exclusion criteria: Children who answered “yes” to any of the questions in the physical activity readiness questionnaire; had a history of cardiac, metabolic, renal, musculoskeletal, or sleep disorders; had a history of oral corticosteroid medication within the past 3 weeks [21]; had a history of 2 courses of oral corticosteroid medications in the past year indicative of poor asthma control; had a history of intensive care unit admission or intubation in the past 5 years; scored 4 or higher on the Tanner pubertal stage self-assessment questionnaire (i.e., late puberty) [22]; or had an FEV_1_ < 75% predicted to help reduce the risk of severe bronchoconstriction during exercise [23,24].

Height was measured using a stadiometer (Charder Medical, Taichung City, Taiwan) with children standing upright. Weight was measured with a weighing scale (Health o meter, Neosho, MO, USA). A pulmonary function test was completed for all children with details provided below. On a separate study visit, albuterol was administered before they completed a CPET. Spirometry measures were performed before albuterol, after albuterol, and within 2 min of terminating exercise. Children were instructed to not consume a heavy meal 2 h before the visits and avoid any caffeine consumption or heavy exercise for 24 h before each visit, which allowed for standardization of testing [25].

### 2.1. Pulmonary Function Test

Children were instructed to hold all asthma medications for 24 h [26]. Spirometry was performed using standard pulmonary function equipment (V62J body plethysmograph, Vyaire Medical, Yorba Linda, CA, USA) according to the guidelines of the American Thoracic Society [27]. Predicted values for spirometric outcome variables were based on norms of the Global Lung Initiative [28].

### 2.2. Cardiopulmonary Exercise Test (CPET)

**Figure 1:**
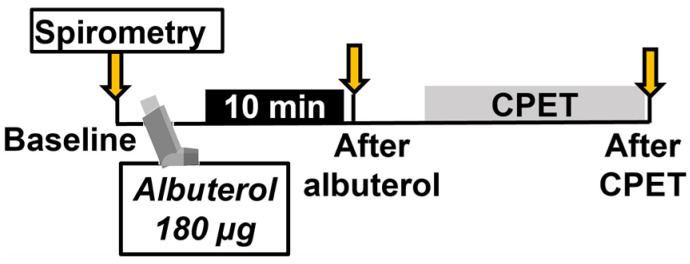
Study protocol. CPET: cardiopulmonary exercise test.

Children were instructed to take their asthma medications as prescribed. Spirometry was performed at the beginning of the visit to establish baseline lung function. A dose of 180 μg of albuterol was administered using a spacer and holding chamber (OptiChamber Diamond, Philips Respironics, Monroeville, PA, USA) [15]. Children were asked to actuate the medication into the spacer device, inhale, and hold their breath for 10 s. The second puff was given in the same way, 50 s later. Albuterol has been known for many years as a well-tolerated bronchodilator. Common side effects such as sore throat, cough, palpitations, shakiness, nausea, increased blood pressure, dizziness, and heartburn are temporary and resolve on their own. The half-life of albuterol is approximately 6 h. Children repeated spirometry 10 min after albuterol administration. Pre- and post-albuterol spirometry was completed before exercise began (Figure 1).

Children completed an incremental exercise test to exhaustion on a cycle ergometer (Viasprint 150P, Bitz, Germany) for the assessment of $\dot{V}$O_2max_. The initial work rate (WR) was set at 20 W for three minutes and was increased every minute by 10 W. Minute ventilation ($\dot{V}$_E_) and gas exchange ($\dot{V}$O_2_ and $\dot{V}$CO_2_) were measured continuously using a metabolic measurement system (Vmax Encore 29C, Vyaire Medical, Yorba Linda, CA, USA). Heart rate and pulse oxygen saturation were recorded every minute (Nellcor PM1000N, Medtronic, Minneapolis, MN, USA). Electrocardiogram (GE Case V6.61, Milwaukee, WI, USA) was observed continuously, and blood pressure was measured every minute (Tango, SunTech Medical, Morrisville, NC, USA). Ratings of perceived exertion (RPE) and breathlessness (RPB) were assessed every minute. Children repeated spirometry within 2 min of terminating the exercise test.

### 2.3. Estimated Ventilatory Capacity

Estimated maximal tidal volume (FVC/2) and total respiratory cycle time (2 × isoFET_25–75_) were incorporated into the following equation (FVC/2) × (60/(2 × isoFET_25–75_)) to calculate estimated ventilatory capacity [11]. To limit confounding due to changes in FVC post-albuterol [29], FET_25–75_ estimates for “after albuterol” and “after incremental exercise” maximal expiratory flow–volume (MEFV) loops from spirometry were based on the same span of absolute lung volumes at which FET_25–75_ was measured at “baseline” (i.e., isovolume). Isovolume FET_25–75_ was denoted as isoFET_25–75_. These calculations were based on the assumption that albuterol does not affect total lung capacity [30].

### 2.4. Breathing Reserve

Breathing reserve was calculated as estimated ventilatory capacity—measured maximum $\dot{V}$_E_/estimated ventilatory capacity × 100. Ventilatory limitation was defined as a breathing reserve of <15% [5].

### 2.5. Statistical Analysis

Data were reported as means ± standard deviations (S.Ds.) unless otherwise specified. In the absence of published data on changes in FET_25–75_ in response to albuterol, we used data on changes in FEV_1_ after 100µg of albuterol in asthmatic patients from Milanese et al. [31] for our power calculations. We estimated that a sample size of 13 participants would detect a significant change in outcome measures at 80% power (α = 0.05, effect size Cohen’s d = 0.86) assuming a correlation of 0.50 between pre- and post-measures. Differences in ventilatory capacity, breathing reserve, and pulmonary function variables between baseline, after albuterol administration, and after the CPET were detected with repeated measures mixed models with Bonferroni post hoc corrections. Pearson correlations were used to examine associations between variables. Analyses were performed using SAS 9.4 (Cary, NC, USA). IBM SPSS Statistics (v28, Armonk, NY, USA) was used to investigate frequency differences (Chi-square test with z-test for post hoc differences). Data were checked for normality with Shapiro–Wilk tests. Nonparametric tests were utilized as needed to meet analysis assumptions. Alpha was set at *p* < 0.05.

## 3. Results

**Figure 2:**
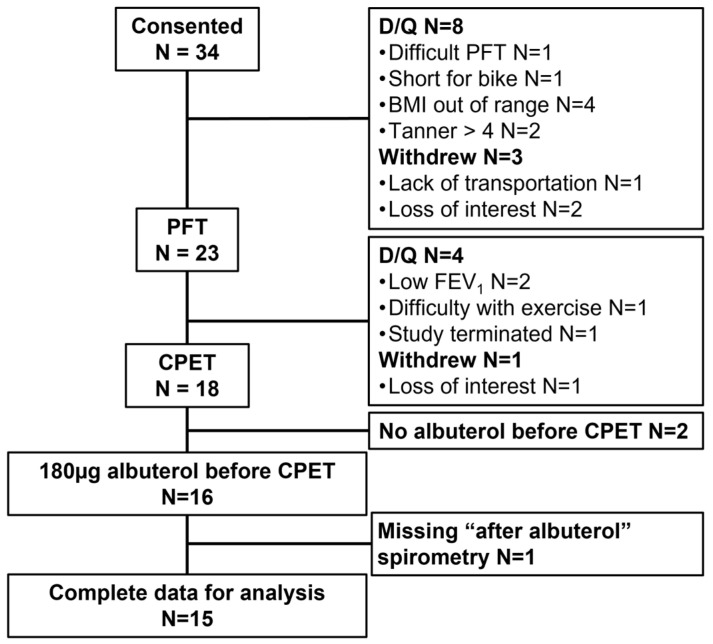
Participant enrolment and retention with reasons for disqualification, withdrawal, and removal from final analysis. PFT: pulmonary function test; CPET: cardiopulmonary exercise test; D/Q: disqualified; BMI: body mass index; FEV_1_: forced expiratory volume in 1 s.

Thirty-four children were enrolled in the study. Twelve children were disqualified, four withdrew, and two completed the maximal exercise test without albuterol pretreatment; data from these individuals were not included in the analyses (Figure 2). We were unable to obtain MEFV loops after albuterol pretreatment for one participant. Thus, data from *n* = 15 children were available for analysis.

Descriptive characteristics are reported for 15 children including 3 with obesity and 12 without obesity (Table 1). All children had relatively well-preserved lung function. Two children had a maximal $\dot{V}$O_2_ that was below 85% of the predicted value, indicative of reduced cardiorespiratory fitness [5].

A 180 µg dose of albuterol increased estimated ventilatory capacity and mid-flows (i.e., FEF_25–75_) by 13% and 23%, respectively, and decreased isoFET_25–75_ by 15% (Table 2). FVC itself was not affected by albuterol. Further, the additional stimulus provided by the CPET increased estimated ventilatory capacity, mid-flows, and FEV_1_ beyond the increase noted with albuterol (Table 2). FVC was not affected by albuterol or exercise.

**Figure 3:**
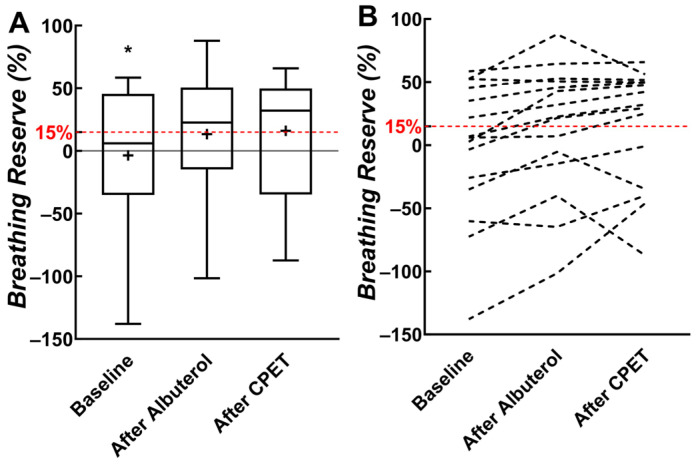
(**A**) Box plots of breathing reserve from baseline, after albuterol, and after cardiopulmonary exercise testing (CPET). (**B**) Individual participant data. The 15% threshold for breathing reserve represents ventilatory limitation and is indicated with a dashed red horizontal line. * Significant difference between baseline and other measures. “+” indicates mean value.

Nine out of fifteen children (60%) were ventilatory limited (i.e., breathing reserve < 15%) when maximal $\dot{V}$_E_ was compared with estimated ventilatory capacity at baseline (Figure 3). Fewer children (*n* = 6; 40%) had a ventilatory limitation when maximal $\dot{V}$_E_ was compared with estimated ventilatory capacity after albuterol was administered. Five children (33%) were classified as ventilatory limited when maximal $\dot{V}$_E_ was compared with estimated ventilatory capacity after the CPET. Frequency differences in the percentage of children who were ventilatory limited or not based on comparisons with ventilatory capacity (at baseline, after albuterol, and after CPET) did not reach statistical significance (*p* = 0.310).

RPB at maximal exercise was correlated with increased maximal *f*_B_ and reduced maximal inspiratory time (Table 3). RPB was also significantly correlated with reduced breathing reserve.

## 4. Discussion

To the best of our knowledge, this is the first study to show that (1) albuterol administered before an incremental exercise test increases mid-expiratory flows leading to a 12.8% increase in estimated ventilatory capacity in children with asthma; (2) the albuterol-induced improvement in mid-expiratory flows occurs in the absence of any significant changes in maximal expiratory flows or FEV_1_; (3) there is an additional 11.7% increase in ventilatory capacity after the CPET suggestive of an additive or synergistic bronchodilator effect of combining albuterol and exercise; and (4) higher mid-expiratory flows may predict lower dyspnea ratings at maximal exercise.

The 12.8% increase in ventilatory capacity with 180 µg albuterol occurred through improvements in mid-expiratory flows and the accompanying reduction in FET_25–75_. Using the same methodology, Wilhite et al. [13] showed that 360 µg of albuterol increased ventilatory capacity by 15.8% in nonasthmatic children with and without obesity. Whether the dose of 180 vs. 360 µg of albuterol influences ventilatory capacity remains to be explored. We used 180 µg because it is the typical dose prescribed to prevent exercise-induced bronchoconstriction.

The CPET appeared to enhance the effect of albuterol in improving maximal and mid-expiratory flow rates as indicated by increases in ventilatory capacity, FEV_1_, and isoFEF_25–75_. These findings are consistent with previous reports in adults with asthma [31,32,33]. Milanese et al. reported an additive bronchodilator effect of albuterol plus CPET in adults with mild-to-moderate asthma [31]. Similarly, 200µg of albuterol in conjunction with high-intensity interval exercise resulted in a 15% increase in FEV_1_ and ~35% increase in FEF_25–75_ when compared with the control condition in adult asthmatics [32]. Rossman et al. reported a 10% increase in FEV_1_ and a 27% increase in FEF_25–75_ after 360µg of albuterol and an exercise performance test at 85% of peak work rate in adult asthmatics [33]. The authors reported reduced exercise ventilatory limitations when exercise was completed after albuterol compared with exercise performed without prophylactic albuterol.

Exercise increases sympathetic activity and circulating catecholamines including norepinephrine [34], which is an agonist for β_2_-adrenergic receptors [35]. Stimulation of β_2_-adrenergic receptors located on the airway smooth muscle increases levels of cyclic adenosine monophosphate, which results in airway smooth muscle relaxation and bronchodilation [35]. Furthermore, reduced parasympathetic activity during exercise results in lower stimulation of M2 and M3 muscarinic receptors on the airway smooth muscle, which are responsible for bronchoconstriction [36]. Thus, the modulation of autonomic function by exercise could explain the mechanism of exercise-induced bronchodilation.

Because ventilatory capacity increased after albuterol and CPET, breathing reserve also increased from −3.6% to 16.1% in our participants. Of the nine children who would be classified as “ventilatory limited” using pre-albuterol baseline ventilatory capacity, four were found to have an adequate breathing reserve when the ventilatory capacity was estimated using post-CPET spirometry. Regardless, approximately 33% of children experienced a limited breathing reserve at maximal exercise despite albuterol and the bronchodilatory effects of exercise. Some children had a “negative” breathing reserve because we used a fixed mid-flow-based estimate to calculate ventilatory capacity. This method cannot account for dynamic hyperinflation during exercise, which would allow children to increase flows more than would be possible in the mid-expiratory flow range and result in levels of *f*_B_ and ventilation that exceed the estimated ventilatory capacity.

RPB at maximal exercise was strongly associated with higher *f*_B_. Thus, alterations in breathing patterns can contribute to dyspnea on exertion. We also observed a moderate negative association between RPB and post-albuterol FEF_25–75_, which suggests that albuterol-facilitated increases in mid-expiratory flows and subsequent increases in ventilatory capacity could decrease exertional dyspnea among asthmatic children. Our study was not designed to investigate whether albuterol decreases dyspnea ratings during incremental exercise testing or whether it can preclude a symptom-limited exercise test.

Short-acting β agonists like albuterol are strongly recommended with high-quality evidentiary support for the prevention of exercise-induced bronchoconstriction in patients who experience exercise-induced bronchoconstriction by the American Thoracic Society [15]. Albuterol is recommended to be administered 15 min before exercise [15]. Our study adds to the existing understanding of the role of albuterol because it offers insight into how albuterol administered before a CPET can increase ventilatory capacity in children with mild asthma. As such, administering albuterol before a CPET could serve a dual role: increasing ventilatory capacity even in patients who may not experience exercise-induced bronchoconstriction and potentially preventing exercise-induced bronchoconstriction for a subset of patients who are at risk.

Strengths and limitations: This study was not designed to directly compare the effects of albuterol vs. no albuterol before incremental exercise testing on exercise capacity. Our goal was to estimate the effect of albuterol on ventilatory capacity. We expect that not giving albuterol would have no effect on pre-exercise airway function, but that any exercise-related bronchodilation could still occur. However, bronchoconstriction could still occur in the absence of albuterol in response to incremental exercise testing [37]. This study was also limited by the high disqualification and dropout rates, which highlights the difficulty of completing physiological studies in pediatric patient populations. Some disqualifications were unavoidable based on study inclusion/exclusion criteria. However, an upfront and clear explanation of study commitment, frequent reminders to parents, and offering incentives that reflect time and effort did eventually help with recruitment and retention. The relatively small sample size including three children with obesity and twelve children without obesity precluded an analysis of data based on obesity status. Removing the children with obesity from the analyses did not change our results. Furthermore, Wilhite et al. [13] reported similar increases in ventilatory capacity between nonasthmatic children with and without obesity after albuterol administration. Therefore, the effect of obesity on post-albuterol mid-expiratory flows and ventilatory capacity may be negligible in the context of relatively normal maximal expiratory flows. Regardless, because obesity has a significant effect on breathing mechanics, future studies could incorporate a larger sample size including obese and nonobese asthmatic children to allow for the analysis of outcome measures based on obesity status in the context of asthma. We had a low number of female children, which precluded an analysis of data based on sex. Because sex-related differences in lung development exist at birth and persist through adulthood and could affect respiratory responses to albuterol and exercise, future projects need to investigate sex differences. Future studies may also consider incorporating impulse oscillometry, which is a simple method of assessing airway resistance in central and peripheral airways [38] to provide more information on the changes in central and peripheral airway function in response to albuterol and CPET. Finally, we recruited children from a pediatric pulmonology clinic where spirometry was completed on all asthmatic children during clinic visits, which is a strength of this study.

## 5. Conclusions

Albuterol and exercise can improve ventilatory capacity in children with asthma. Administering albuterol before a CPET could potentially mitigate the risk of ventilatory limitations among asthmatic children with relatively normal maximal expiratory flows, prevent early termination of testing or a symptom-limited test, and allow for an accurate assessment of functional capacity. This knowledge is relevant to clinicians performing CPETs in children with asthma.

## Figures and Tables

**Table 1:** Participant demographics, medication use, spirometry, and cardiopulmonary exercise variables.

	*n* = 15
Age	10.6 ± 0.9
Sex (M,F)	11,4
Tanner (Stages 1,2,3)	6,5,4
Height (cm)	143.6 ± 6.6
Body mass (kg)	41.1 ± 12.1
BMI (kg/m^2^)	19.73 ± 4.73
BMI (Z score)	0.59 ± 0.97
BMI (% ile)	64.86 ± 25.29
*Medications*	
Inhaled steroids (*n*)	7
LABA (*n*)	2
Leukotriene Receptor Antagonist (*n*)	7
LAMA (*n*)	1
SABA only (*n*)	3
No meds (*n*)	1
*Spirometry*	
FVC (L)	2.70 ± 0.41
FVC (% pred)	112.55 ± 13.70
FEV_1_ (L)	2.11 ± 0.35
FEV_1_ (% pred)	101.90 ± 16.79
FEV_1_/FVC	78.20 ± 6.59
FEF_25–75_ (L/s)	2.00 ± 0.60
FEF_25–75_ (% pred)	81.70 ± 25.79
PEF (L/s)	4.51 ± 0.82
PEF (% pred)	89.44 ± 13.15
*Maximal exercise*	
Work rate (W)	95 ± 20
$\dot{V}$O_2_ (L/min)	1.51 ± 0.38
$\dot{V}$O_2_ (ml/kg/min)	38.33 ± 10.11
$\dot{V}$O_2_ (% pred)	103.03 ± 18.15
$\dot{V}$CO_2_ (L/min)	1.69 ± 0.43
RER	1.11 ± 0.06
$\dot{V}$_E_ (L/min)	53.6 ± 14.5
V_T_ (L)	1.12 ± 0.24
*f*_B_ (/min)	49 ± 11
V_T_ (%FVC)	41.49 ± 6.16
Heart rate (bpm)	186 ± 14
SpO_2_ (%) *	97.9 ± 1.4
RPB (Borg 0–10 scale)	5.3 ± 2.8
RPE (Borg 6–20 scale)	16.5 ± 1.9

Abbreviations: M, male; F, female; BMI, body mass index; LABA, long-acting beta-agonist; LAMA, long-acting muscarinic antagonist; SABA, short-acting beta agonist; FVC, forced vital capacity; FEV_1_, forced expiratory volume in 1 s; FEF_25–75_, forced expiratory flow between 25% and 75% of FVC; PEF, peak expiratory flow; $\dot{V}$O_2_, oxygen consumption; $\dot{V}$CO_2_, carbon dioxide production; RER, respiratory exchange ratio; $\dot{V}$_E_, minute ventilation; V_T_, tidal volume; *f*_B_, breathing frequency; SpO_2_: pulse oxygen saturation. RPB, ratings of perceived breathlessness; RPE, ratings of perceived exertion. * *n* = 14; poor SpO_2_ signal during exercise for one subject.

**Table 2:** Respiratory responses at baseline, after albuterol administration, and after the cardiopulmonary exercise test (CPET).

*n* = 15	Baseline	After Albuterol	After CPET	*p* Value	Post Hoc
Ventilatory capacity (L/min)	60.9 ± 22.0	68.7 ± 21.2	75.8 ± 25.6	0.0003	a,b,c
Breathing reserve (%)	−3.6 ± 55.1	13.4 ± 51.3	16.1 ± 46.6	0.0034	a,b
FVC (L)	2.71 ± 0.46	2.70 ± 0.44	2.75 ± 0.45	0.3673	
FVC (% pred)	113 ± 15	113 ± 13	115 ± 14	0.4007	
FEV_1_ (L)	2.15 ± 0.42	2.21 ± 0.41	2.31 ± 0.48	0.0005	b,c
FEV_1_ (% pred)	104 ± 20	107 ± 19	112 ± 21	0.0138	b,c
isoFEF_25–75_ (L/min)	2.05 ± 0.74	2.43 ± 0.73	2.69 ± 0.92	<0.0001	a,b,c
isoFEF_25–75_ (% pred)	84 ± 32	100 ± 31	110 ± 39	0.0024	a,b,c
PEF (L/min)	4.50 ± 1.17	4.59 ± 0.80	4.96 ± 0.79	0.1079	
isoFET_25–75_ (s)	0.75 ± 0.27	0.63 ± 0.29	0.62 ± 0.25	0.0088	a,b

Abbreviations: isoFEF_25–75_, mean forced expiratory flow between 25 and 75% of FVC; PEF, peak expiratory flow; isoFET_25–75_, isovolume forced expiratory time between 25 and 75% of FVC. a: baseline vs. albuterol; b: baseline vs. CPET; c: albuterol vs. CPET.

**Table 3:** Correlations between ratings of perceived breathlessness (RPB) at maximal exercise and ventilatory parameters (*n* = 15).

	RPB: Pearson’s *r*	*p* Value
Max T_tot_	−0.739	0.002
Max *f*_B_	0.736	0.002
Max T_i_	−0.697	0.004
Breathing reserve (%)		
After incremental exercise	−0.592	0.020
Baseline	−0.589	0.021
After albuterol	−0.475	0.074
isoFEF_25–75_ (after albuterol)	−0.539	0.038

T_tot_, total respiratory cycle time; *f*_B_, breathing frequency; T_i_, total inspiratory time;isoFEF_25–75_, isovolume forced expiratory flow between 25 and 75% of FVC.

## Data Availability

The data presented in this study are available on request from the corresponding author.
